# Integrating natural language processing and genome analysis enables accurate bacterial phenotype prediction

**DOI:** 10.1093/nargab/lqaf174

**Published:** 2025-12-29

**Authors:** Daniel Gómez-Pérez, Alexander Keller

**Affiliations:** Cellular and Organismic Networks , Faculty of Biology, Ludwig Maximilian University of Munich, 82152 Munich, Germany; Cellular and Organismic Networks , Faculty of Biology, Ludwig Maximilian University of Munich, 82152 Munich, Germany

## Abstract

Understanding microbial phenotypes from genomic data is crucial for studying co-evolution, ecology, and pathology. This study presents a scalable approach that integrates literature-extracted information with genomic data, combining natural language processing and functional genome analysis. We applied this method to publicly available data, providing novel insights into predicting microbial phenotypes. We fine-tuned transformer-based language models to analyze 3.83 million open-access scientific articles, extracting a phenotypic network of bacterial strains. This network maps relationships between strains and traits such as pathogenicity, metabolism, and biome preference. By annotating their reference genomes, we predicted key genes influencing these traits. Our findings align with known phenotypes, reveal novel correlations, and uncover genes involved in disease and host associations. The network’s interconnectivity provides deeper understanding of microbial communities and allowed identification of hub species through inferred trophic connections that are difficult to infer experimentally. This work demonstrates the potential of machine learning for uncovering cross-species gene–phenotype patterns. As microbial genomic data and literature expand, such methods will be essential for extracting meaningful insights and advancing microbiology research. In summary, this integrative approach can accelerate discovery and understanding in microbial genomics. Ultimately, such techniques will facilitate the study of microbial ecology, co-evolutionary processes, and disease pathogenesis to an unprecedented depth.

## Introduction

The quest to decode the intricate relationship between an organism’s genome and its phenotype has been a cornerstone of biological research since the discovery of DNA as the source of heritable material [1–3]. Contrary to initial expectations, the link between phenotype and genotype remains complex and challenging to unravel as there is not a one-to-one relationship between phenotypes and genes [4]. Rather, most phenotypes are polygenic, depending on multiple genes for expression [5, 6]. Adding to this complexity, the genomic context of a gene and epigenetic factors also play a role in the expression of distinct phenotypes from the same genomic material [7, 8]. Despite the increasing wealth of publicly available genomic data, the challenge of predicting accurate microbial phenotypes from the relatively small and low-complexity bacterial genomes persists, presenting a critical bottleneck in fields ranging from epidemiology to environmental science. This study introduces a novel approach to this enduring problem, leveraging the potential of natural language processing (NLP) and machine learning.

Despite hundreds of thousands of high-quality sequenced prokaryotic genomes being available in public databases such as NCBI, a significant challenge remains in the form of limited accompanying metadata [9]. The latter, which includes information such as environmental and growth conditions, as well as phenotypic characteristics, is crucial for a comprehensive understanding of the genomic data. As a result, genetic data, while extensive, cannot be fully leveraged or properly interpreted without this contextual information. This limitation restricts the potential for meaningful insights in areas such as host interaction, metabolic assessment, and environmental adaptability of bacteria. Although databases exist that tackle this problem, they are mostly focused on metabolite usage and lack more complex phenotype annotations [10, 11]. To address this gap, there is an urgent need for more systematic and thorough documentation of phenotypic traits alongside genomic sequences as well as more efficient tools to synthesize and summarize them [12]. Such advancements would significantly enhance our ability to harness the full potential of genomic datasets, leading to more accurate predictions of bacterial behavior and responses under varying conditions.

Recent progress in NLP, including the development and high popularity of large language models (LLMs) based on transformer architecture, has opened new avenues in biological research [13, 14]. Specifically, models based on the BERT architecture pre-trained on scientific texts such as SciBERT [15] and BioBERT [16] have been fine-tuned to perform tasks such as named entity recognition (NER) and relation extraction (RE) to obtain meaningful information from literature. Compared to foundational models such as ChatGPT, these specific models have been shown to perform better in benchmarks for data mining tasks [17]. More recently, BioLinkBERT [18], a BERT-type model trained on hyperlinks connecting scientific articles, has shown the best overall performance on the Biomedical Language Understanding & Reasoning Benchmark, BLURB [19]. BLURB includes datasets for NER and RE tasks such as NCBI-disease [20] and drug–drug interactions [21], respectively. These are straightforward problems with a small number of different types of entities and relations that serve as benchmark and proof-of-concept. However, complex multimodal implementations leading to novel insights are lacking in the literature. In particular, the problem of phenotype–genotype correlations has not yet been tackled using such approaches outside the medical domain. Encoder-only transformer models such as BioLinkBERT offer a promising solution to the long-standing challenge of linking genotypic information with phenotypic traits by easing literature mining and interpretation.

This work aims to bridge the gap between genomic datasets and limited phenotypic metadata. We leverage the power of NLP, specifically fine-tuned BioLinkBERT models, to systematically extract information from the vast corpus of PubMed Central (PMC) and paint a picture of the microbial phenotype landscape in scientific literature. By integrating this extracted knowledge with functional genome annotations from public databases, we investigate correlations between bacterial phenotypes and their underlying genetic makeup. We additionally show several examples of using this network to extract biological insights, including the inference of microbe–microbe interactions and gene–host correlations. This study expands the applications of cutting-edge NLP techniques in biology, ultimately leading to new insights and a deeper understanding of microbial life.

## Materials and methods

### Data acquisition and model preparation

#### Corpus database

The PMC database was selected as the source for the literature corpus due to its comprehensive and up-to-date collection of open-access scientific articles together with license information for data mining. Article data were retrieved from the PMC release dated 26 June 2025, including all commercial and noncommercial open-access datasets. After filtering out journals not specifically related to biology and microbiology, sentence tokenization (breaking text into sentences) and section classification were performed using the R package tinypmc. The entity annotation in the training dataset was designed to represent the frequency of strain-phenotype information in the literature since the approach at the beginning of the manual annotation was to randomly select sentences containing exact strain matches. However, this shifted as we added more sentences to the annotated dataset to improve the models.

Sentences unlikely to contain strain descriptions were discarded, including acknowledgements, author declarations, data availability statements, and legends. No sentence length cutoff was applied, and sequences longer than the context window of the BERT models (512 tokens) were truncated by the tokenizer during inference. To improve the context window of the predictions, short paragraphs of <512 characters were included in full. While single sentences were used in the annotation dataset, models showed successful inference across sentences at the paragraph level. Additionally, duplicated sentences and those in languages other than English were discarded based on predictions from the lingua-py package (confidence in English language $>$40%). We replaced the hyphen in all hyphenated words with a space to avoid token splitting.

#### Entities and relations

We used two modeling tasks: NER, which identifies words belonging to predefined entities and RE, which predicts at the sentence level whether a relation holds between a given entity pair.

We define entities as named concepts detected by the NER models. Microbial phenotype information was extracted as entity terms, which are defined as the values of these entities. Entities belonged to the following categories: taxonomic (STRAIN, SPECIES, ORGANISM), phenotypic (PHENOTYPE, EFFECT, DISEASE), and environment/molecule-related entities (MEDIUM, ISOLATE, COMPOUND). STRAIN and SPECIES exclusively included properly characterized bacterial strains and species. All other taxonomic classifications, including fungal strains or animal species, were grouped under ORGANISM. The phenotypic entities were classified into PHENOTYPE, including for example the shape of bacteria or trophic characteristics. EFFECT contained a variety of entity terms indirectly related to effects caused by strains that did not fit into the PHENOTYPE entity. DISEASE contained the names of diseases or pathological signs. COMPOUND included organic and inorganic molecules, such as common metabolites, metals, and antibiotics. Finally, MEDIUM was related to the growth medium of bacteria in laboratory settings, and ISOLATE to the type of sample from which the particular bacterial strain was acquired.

We define relations as broad linking categories that connect the previous entities. We designate the following relations: interaction with environment (GROWS_ON, INHABITS), metabolic interaction (PRODUCES, DEGRADES, RESISTS), biological phenotype (PRESENTS, ASSOCIATED_WITH, PROMOTES), and interaction with other organisms (SYMBIONT_OF, INFECTS, INHIBITS). SYMBIONT_OF relation, being a subcategory type of INHABITS relation, had both labels. The relation types were not restricted to particular entities but followed logical sense, resulting in cases where the same relation type connected different kinds of entities (i.e. INHIBITS, was both used to connect STRAIN and ORGANISM as well as COMPOUND and STRAIN). However, in some cases the entity was connected through an exclusive relation type (i.e. GROWS_ON, for MEDIUM). For clarity, we refer to specific relations in this document by specifying first the two entities linked by the relation type in the direction of the connection with a dash, followed by a colon and the relation type (i.e. sourceENTITY-targetENTITY:RELATIONTYPE).

#### Annotation

Initially, we performed a manual annotation of entities and their relations on a set of randomly selected sentences that contained popular strains matched by their exact strain name as found in the literature corpus. With these initial annotations, we trained preliminary NER and RE models and ran them on a small subset of the full corpus. In order to improve the models, we iteratively selected and annotated new sentences containing predicted entities and relations in cases where they differed widely from the ground truth. In total, we annotated 3979 sentences. The annotated dataset included sentences with both presence or absence of each of the entities. Since the base rate for the presence of strains in random sentences from the literature is relatively low, a large number of nonentity-containing sentences ($\sim$35.1%) were included to prevent false positives. Common variations of some of the most abundant entity terms after NER prediction were added by manual curation to improve downstream analyses.

We complemented the training datasets for both NER and RE models with an augmented dataset for positively labeled entities (i.e. sentences containing the target entity) to counteract the disparity in positive versus negative labels (sentences without the target entity) in the annotated dataset. To reduce class imbalance, for each NER entity with $N_{\mathrm{pos}}$ positives and $N_{\mathrm{neg}}$ negatives we added $N_{\mathrm{aug}} = \max (0,\, N_{\mathrm{neg}}-N_{\mathrm{pos}})$ synthetic positive sentences (created by randomly swapping STRAIN terms from the curated catalog while leaving the rest of the sentence unchanged). For each RE relation we used a lighter augmentation rate to limit runtime: we added one-fifth of this gap, i.e., $N_{\mathrm{aug}} = \left\lceil 0.2\, (N_{\mathrm{neg}}-N_{\mathrm{pos}})\right\rceil$.

#### Model fine-tuning

We fine-tuned a base BioLinkBERT-large model (24 encoder layers, 16 attention heads, and 340M parameters) for each of the entities with the NER task, resulting in nine separate single-entity token-classification models. A stratified split (same proportion of labeled sentences on each set) based on all NER annotations was performed, resulting in 65%, 17.5%, and 17.5% of the total sentences in the annotated dataset, corresponding to training, validation, and testing, respectively. For each entity, a Beginning-Inside-Outside (BIO) encoding was applied to the training sentences and each of the 9 models was fine-tuned for at least 20 epochs with early stopping of 5 epochs (after no improvement in loss for 5 consecutive epochs, training stops and best model is selected) at a learning rate of $2 \times 10^{-5}$. BIO encoding consists of three label classes $C$ which take class $c$ for each token $t$ in a sentence ($c \in \lbrace B,I,O\rbrace$). If the token is at the start of the entity, its class is $B$ and each continuing token is $I$, all others outside of the entity are labeled as $O$. The best models were selected based on the minimization of cross-entropy loss $L$ in the evaluation set for each entity. For the set $N$ of sentences in the training dataset, the loss was calculated as:


\begin{eqnarray*}
L = - \frac{1}{N} \sum _{i = 1}^{N} \sum _{t = 1}^{T_i} \sum _{c = 1}^{C} \delta (y_{it}, c) \cdot \log \hat{p}_{itc}
\end{eqnarray*}

where $T_i$ is the number of tokens in the $i$-th sentence and $y_{it}$ is the true label for the $t$-th token in the $i$-th sentence. The $\delta (y_{it}, c)$ corresponds to the Kronecker delta function, and is 1 if the label $y_{it}$ equals class $c$, and 0 otherwise. The AdamW optimizer was employed for gradient descent as implemented in the Hugging Face transformers package [22].

For the evaluation of NER models we followed two strategies, exact match which only considers exact token matches for evaluation of precision ($P$) and recall ($R$):


\begin{eqnarray*}
P = \frac{COR}{TP + FP}; R = \frac{COR}{TP + FN}
\end{eqnarray*}

where, $TP$ is true positives, $FP$ false positives, $FN$ false negatives, and $COR$ is the number of complete overlaps (exact matches). As well as partial matching, which takes into account overlap in the tokens corresponding to an entity ($PAR$, the number of partial, nonexact overlaps):


\begin{eqnarray*}
P = \frac{COR+0.5 \times PAR}{TP + FP}; R = \frac{COR+0.5 \times PAR}{TP + FN}
\end{eqnarray*}

Unless otherwise noted, we report strict, exact-span precision, recall, and F1 for cross-paper comparisons; we use partial/overlap (lenient) matching only as a within-study diagnostic to quantify boundary near-misses for multi-token biomedical mentions, as employed in [23–26].

Regarding RE models, we prepared a dataset for each relation type consisting of sentences annotated with binary labels indicating the presence and the absence of the relation between the specified entity terms. We split all pairwise relations into 60% training, 20% evaluation, and 20% testing, performing stratification in this case per individual relation, due to disparities in entity distributions of the dataset. For the training of RE models, we used a similar strategy as for the NER models, but optimizing instead for a higher F1 score in the evaluation dataset, the harmonic mean of precision and recall:


\begin{eqnarray*}
F1 = 2 \frac{P \cdot R}{P + R},
\end{eqnarray*}

for a total of at least 15 training epochs with early stopping and a learning rate of $3 \times 10^{-5}$. All training was done on four Nvidia A100 Tensor Core GPUs (80GB memory) in parallel using the PyTorch backend [27].

For the prediction pipeline, we initially used the STRAIN model to annotate all relevant sentences from the filtered corpus in the PMC database. In the NER annotation, we considered a positive hit when the prediction score was 0.5 or higher ($p(t_i, c) \ge 0.5$) after aggregation of consecutive tokens ($T_c = (t_k, t_{k+1}, \ldots , t_n)$). Token aggregation was performed using the max aggregation score from the Hugging Face transformers package, which results in the entity assignment with the maximum score for a series of $T_c$ tokens when $c \in \lbrace B,I\rbrace$:


\begin{eqnarray*}
p(T_c, c) = \max _{t_i \in T_c} p(t_i, c),
\end{eqnarray*}

28]. On the resulting positive sentences for the presence of a STRAIN entity, we performed predictions with the remaining eight NER models. Finally, we applied the RE models for extracting relations on sentences with at least two entities, one of which corresponding to a strain, by pairing all combinations of STRAIN with other entities within the same sentence. We chose those with a score over 0.5 for a positive relation and compiled the annotations. The threshold of 0.5 in the NER and RE predictions was chosen due to standard practice in machine learning, and led to balanced precision and recall. Due to the similar prevalence of entities in the training and corpus, the cutoff was left in place.

For visualizing the learned representations of the models, we used the UMAP dimensionality reduction algorithm [29]. For sentences with either presence of at least a positive entity in the NER models or a positive relation with RE, we applied UMAP on the per-sentence mean token outputs from the last hidden layer, resulting in a 1024-dimensional vector for each sentence. Default UMAP parameters were used except for n_neighbors$=$3 and min_dist$=$0.001. This allowed better visualization of the local differences as opposed to the global picture, which was more informative in this case.

### Network analysis

#### Network construction

To analyze the interconnectivity between entities and relations, we represented the extracted information as a directed network $G = (V, E)$, where $V$ is the set of nodes representing entities, and $E$ is the set of edges representing the relations between entities. The degree distribution $P(k)$ of the network, where $k$ is the degree of a node (the number of connections it has), was assessed to determine if the network exhibits a scale-free property, which follows a power-law distribution:


\begin{eqnarray*}
P(k) \sim k^{-\alpha },
\end{eqnarray*}

where $\alpha$ is the scaling exponent. Algorithms for the network analyses, including distance metrics and triad assessment, were applied using the Graph-tool and NetworkX packages in Python [30].

#### Community interaction

From the subnetwork constructed of strains and compounds associated with the same environment, we considered two types of negative strain correlations. Either when two different strains degraded the same compound or when a compound produced by one strain was responsible for an INHIBITS relation towards another strain, as an example of resource competition and direct inhibition, respectively. Positive correlations between strains were defined by the degradation of a product by a strain that is produced by another to exemplify cross-feeding, as well as a RESISTS relation of a strain toward a product produced by another strain to represent a resistance-development-type interaction. For each pair of strains in the subnetwork $(s_i, s_j)$, we calculated the interaction weight $w_{ij}$:


\begin{eqnarray*}
w_{ij} = n_{ij}^+ - n_{ij}^-,
\end{eqnarray*}

where $n_{ij}^+$ and $n_{ij}^-$ are the number of positive and negative interactions inferred between strains $s_i$ and $s_j$, respectively. The interaction weight $w_{ij}$ provides a quantitative measure of the overall relationship between two strains, with positive values indicating net positive interactions and negative values indicating net negative interactions.

### Phenotype–genotype correlation analysis

To handle potential biases and disambiguations in the name of the strains, we matched the resulting predicted terms in the STRAIN entity to the StrainSelect reference database, which links different nomenclatures for the same strain, using a custom approach based on Levenshtein string distances [31, 32]. This included first a local distance match to assess partial strain names due to the common presence of abbreviations, followed by a full distance assessment to map complete names. We performed a similar approach on non-STRAIN entities in order to group variations of the same entity, such as plural forms or typos. The representative entity term was chosen based on the largest number of occurrences in the literature.

We downloaded the NCBI RefSeq strains connected to each of the strain matches. For each assembly, we predicted the proteome using the Prodigal prokaryotic annotation tool [33]. We performed *de novo* functional annotation using InterProScan 5.67 based on homology to Pfam families [34, 35]. We then encoded all functional annotations using their abundance (number of copies for an annotation) in that particular assembly as features and grouped all strains that had the same phenotype relation in a matrix.

Enrichment of GO terms was calculated using Fisher’s exact test and correcting $p$-values for multiple testing using false discovery rate as implemented in GOATOOLS [36] at the level of relation. This means that all genes from all strains involved in any instance of a relation were pooled together. GO release 2024-06-17 was employed. Species phylogenies were inferred using the Open Tree of Life API Python package: OpenTree [37].

#### Gradient boosting

After discarding entity terms appearing fewer than three times, we defined a phenotype cluster by grouping all strains sharing a specific relation type. We binarized each phenotype cluster by labeling as 1 if they contained this entity term and 0 otherwise across all sentences in the corresponding relation. We then performed a stratified split to separate the train and test sets (80% and 20%, respectively) for each phenotype cluster. We used a gradient boosting approach for binary classification of each of the entities that were found in 10 or more assemblies from five or more different genera where the most abundant genus is <30% of the total strains, using the XGBoost library [38].

Each model (maximum tree depth of 6, learning rate of 0.3, binary logistic objective function, log loss as evaluation metric and the gradient boosting tree algorithm *gbtree*) was trained using early stopping to mitigate overfitting: the algorithm was allowed to train for a maximum number of 5000 boosting rounds, but training was terminated if the validation loss failed to improve for ten consecutive rounds. Following training, model accuracy was measured on the hold-out test set. For models with accuracy >80%, we extracted the importance gain, corresponding to the average improvement in the training set loss brought by a particular feature. We used this to rank and interpret how relevant the functional features were for the corresponding phenotype labels and correlations.

#### Positive selection analyses

To study the selective pressures of high-importance genes, we selected gene annotations belonging to strains within a specific relation type that had the same annotation. We aligned these corresponding proteins using MAFFT, mapped nucleotide sequences with PAL2NAL to create codon alignments [39, 40]. After inferring the protein maximum likelihood trees with FastTree, we assessed the presence and significance of gene-level positive selection for annotations of each relation type using BUSTED [41, 42]. BUSTED is based on the ratio of nonsynonymous ($d_N$) to synonymous ($d_S$) amino acid changes to infer selective pressure:


\begin{eqnarray*}
\omega = \frac{d_N}{d_S},
\end{eqnarray*}

where $\omega < 1$ indicates purifying selection, and $\omega > 1$ indicates positive selection. BUSTED fits each gene to a constrained null model ($M_0$) that does not allow for positive selection ($\omega \le 1$) and an alternative unconstrained model ($M_1$) that allows for positive selection ($\omega > 1$). The likelihood ratio test (LRT) statistic is given by:


\begin{eqnarray*}
\text{LRT} = 2 \cdot \left( \log L_{M_1} - \log L_{M_0} \right),
\end{eqnarray*}

where $L_{M_0}$ and $L_{M_1}$ are the likelihoods of the null and alternative models, respectively. A significant LRT value suggests evidence for positive selection.

#### BacDive validation

We used the BacDive database [10] as a reference to assess overlap with our literature-derived phenotypes. The BacDive release used was 2024-12-19 and was accessed through their API (v1.0.0). Comparisons were performed at the strain level only using exact NCBI RefSeq genome accession matches, and exclusively comparing manually curated entries from phenotypes, place of isolation and growth media. We performed BacDive matching after entity grouping/error correction and before all downstream analyses.

## Results and discussion

### Phenotype data extraction from literature

#### Annotations

**Figure 1.**
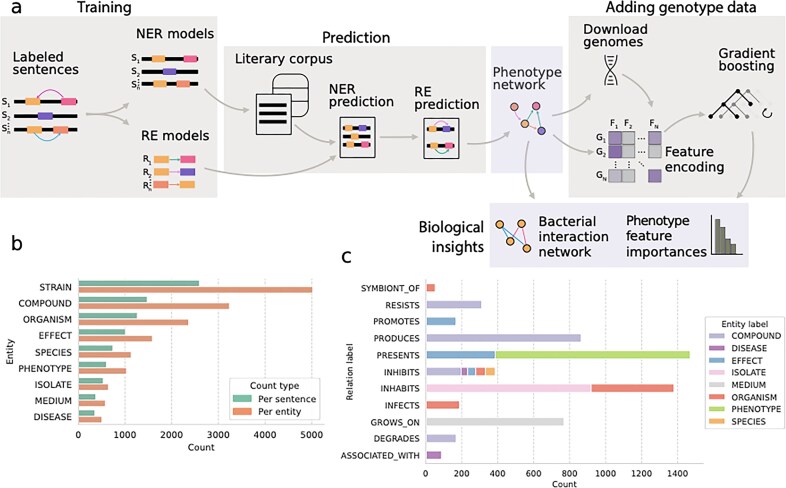
Workflow and training dataset. (**A**) Schematic of the bioinformatics pipeline used in this study. (**B**) Bar chart showing the number of entities annotated either per sentence or over the total dataset. (**C**) Stacked bar chart showing the total number of entity annotations per relation type. The colored bars represent different entities and their size indicates the number of their respective occurrences in the same sentence with a STRAIN entity in the training dataset.

We extracted microbial phenotype information from the literature corpus using the bioinformatics workflow described in Fig. 1A. Out of the 3979 manually annotated sentences, 2596 ($\sim$64.2%) contained at least one STRAIN entity term (Methods 2.1.2), while the total number of individual bacterial strains annotated was about double this figure, with a similar pattern for other entities (Fig. 1B). As expected, most sentences had a single STRAIN entity term, as this was the key entity from which we aimed to extract relational information. The lowest number of entity annotations corresponded to DISEASE, which had 356. However, the number of entity annotations in the annotated dataset was not correlated with the number of relations to STRAIN, but rather related to how phenotype information is described in the literature. For example, the number of relations to STRAIN was larger for ISOLATE (the name of the strain is frequently reported together with where the sample was taken) than to SPECIES, which frequently is reported in a list with other strains, to which no direct relations can be drawn.

Relation types with the highest number of annotations included PRESENTS, PRODUCES and INHABITS (*n*1800, *n*$\sim$1600, and *n*$\sim$1450, respectively), followed by GROWS_ON and INHIBITS with $\sim$850 and $\sim$500 (Fig. 1C). The remainder had between 53 (SYMBIONT_OF) and 360 (INHIBITS) annotated relations. The most abundant same-sentence co-occurring entity with STRAIN included COMPOUND, SPECIES, and ORGANISM, while the least abundant corresponded to MEDIUM and DISEASE, following the same pattern of entity abundances. However, more abundant entities did not necessarily have proportionally more relations to STRAIN. The most abundant relations corresponded instead to MEDIUM and PHENOTYPE, while SPECIES had the least (Supplementary Fig. S2). As mentioned earlier, this relates to patterns of reporting strain information in scientific papers.

#### Accurate prediction across entities

**Figure 2.**
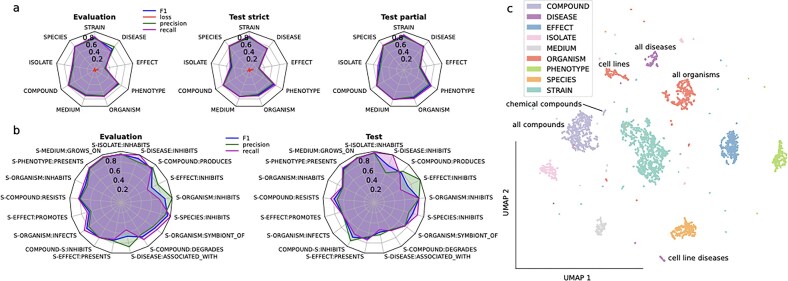
Model performance. (**A**) Overview of NER model training and testing. Performance metrics for the NER models for the evaluation, and test datasets for either strict or partial metrics. (**B**) Overview of the RE model training and testing. Performance metrics for the RE models for either the evaluation or the test datasets; S: STRAIN. (**C**) UMAP representation of sentence output embeddings in the annotated dataset for each of the different NER models. Displayed are only sentences that are positive for the presence of that particular entity. Axes correspond to UMAP dimensions 1 and 2.

The NER models resulted in a combined precision and recall score (F1) of over 70% for most entities in the test (not seen before by the model) and evaluation (used to optimize weights during training) datasets, based on strict matching metrics (Table 1). STRAIN scored the highest across all metrics ($\mathrm{F1}>0.83$, Fig. 2A). The lower performance for EFFECT ($\mathrm{F1} = 0.22$) and ISOLATE ($\mathrm{F1} = 0.32$) in the test dataset may be attributed to the subjective nature of these entities. For the former, these included phenotype descriptions that did not fit classical microbial phenotypes, such as indirect effects on a host. For the latter, these included very specific isolation location descriptions. The number of samples in the training dataset for both these categories may have been too small to capture this diversity, and thus is reflected in the higher loss after training. To isolate the contribution of data augmentation (Methods 2.1.3), we retrained each NER/RE model on identical splits with augmentation disabled. Augmentation yielded small but consistent gains on the test set: +3.3% F1 (NER) and +2.1% F1 (RE).

**Table 1.** Named entity recognition model performance metrics

	Evaluation			Test strict			Test partial		
Entity	F1	Prec	Rec	F1	Prec	Rec	F1	Prec	Rec
STRAIN	0.839	0.813	0.866	0.858	0.836	0.882	0.886	0.862	0.912
SPECIES	0.795	0.795	0.795	0.810	0.802	0.818	0.830	0.822	0.838
ISOLATE	0.578	0.561	0.597	0.472	0.446	0.500	0.642	0.608	0.681
COMPOUND	0.777	0.762	0.792	0.775	0.769	0.780	0.809	0.803	0.815
MEDIUM	0.690	0.672	0.709	0.731	0.731	0.731	0.808	0.808	0.808
ORGANISM	0.717	0.707	0.728	0.686	0.663	0.711	0.754	0.728	0.781
PHENOTYPE	0.705	0.730	0.682	0.735	0.699	0.775	0.781	0.743	0.824
EFFECT	0.278	0.260	0.297	0.255	0.244	0.266	0.422	0.405	0.442
DISEASE	0.710	0.786	0.647	0.723	0.732	0.714	0.795	0.805	0.786

We also applied partial matching metrics on the test dataset, which offered a more lenient criterion for multi-token entities and showed better performance across all entities (overall improvement in F1$=$7.6%, Table 1). Especially notable is the increase in performance for the lowest performing in the strict matching, which highlights a limitation with the exact matching of these longer entities.

Overall, the results corresponded to an accurate prediction across all entities, with strict scores comparable (F10.8) to other applications of fine-tuned BioLinkBERT [17, 18]. Regarding RE models, we found high evaluation metrics overall for successful prediction (Fig. 2B). The F1 score for all 17 models was between 0.6 and 0.9 in all cases in both evaluation and test datasets, except in STRAIN-SPECIES:INHIBITS (Table 2). The higher performance compared to NER prediction may be due to the binary nature of the prediction per sentence, instead of per token. Additionally, the straightforward nature of most relations containing both a STRAIN and other entities resulted in the presence of a relation in more than half the occurrences of STRAIN with other entities (Supplementary Figs S1 and S2). In general, relations with lower abundance in the annotation set correlated to lower metrics in the test set, including two INHIBITS relations, but not in the evaluation set (Fig. 2B). This implies that a larger manual annotation set could lead to more accurate predictions on novel data.

**Table 2.** Relation extraction model performance metrics

	Evaluation			Test		
Relation type	F1	Prec	Rec	F1	Prec	Rec
STRAIN-ISOLATE:INHABITS	0.942	0.949	0.935	0.971	0.964	0.978
STRAIN-MEDIUM:GROWS_ON	0.949	0.941	0.957	0.930	0.938	0.922
STRAIN-PHENOTYPE:PRESENTS	0.909	0.898	0.920	0.892	0.871	0.914
STRAIN-ORGANISM:INHABITS	0.772	0.737	0.812	0.686	0.676	0.696
STRAIN-COMPOUND:RESISTS	0.812	0.796	0.830	0.780	0.722	0.848
STRAIN-EFFECT:PROMOTES	0.642	0.607	0.680	0.667	0.654	0.680
STRAIN-ORGANISM:INFECTS	0.833	0.781	0.893	0.667	0.692	0.643
COMPOUND-STRAIN:INHIBITS	0.800	0.800	0.800	0.792	0.875	0.724
STRAIN-EFFECT:PRESENTS	0.743	0.764	0.724	0.705	0.672	0.741
STRAIN-DISEASE:ASSOCIATED_WITH	0.667	0.875	0.538	0.583	0.636	0.538
STRAIN-COMPOUND:DEGRADES	0.786	0.733	0.846	0.627	0.615	0.640
STRAIN-ORGANISM:SYMBIONT_OF	0.824	0.778	0.875	0.750	0.750	0.750
STRAIN-SPECIES:INHIBITS	0.941	0.889	1.000	0.778	0.700	0.875
STRAIN-ORGANISM:INHIBITS	0.857	1.000	0.750	0.875	0.875	0.875
STRAIN-EFFECT:INHIBITS	0.667	0.800	0.571	0.727	1.000	0.571
STRAIN-COMPOUND:PRODUCES	0.898	0.919	0.877	0.784	0.828	0.744
STRAIN-DISEASE:INHIBITS	0.923	0.857	1.000	0.769	0.625	1.000

The lower accuracies of some NER models likely corresponded to the high variation in the number of annotated entities for training (Fig. 1B), rather than misclassification by the model. Not all entities with low number of annotations correlated with lower prediction (Supplementary Fig. S3). This variability may also be related to the complexity of the particular entity and whether its complexity is captured by the examples in the annotation dataset, which is reflected in the higher cross-entropy loss after training. However, the performance difference of the NER models in the evaluation datasets is unlikely to have a large impact on the final results. Mispredictions at this stage may be filtered out by the RE models, which had overall better metrics (Table 2), and the entity string matching downstream, which selected only strains with a database match, although it may have reduced the number of final relations.

#### UMAP uncovers model representations

To understand how the models predicted the different entities, we visualized the embeddings of the 8 different NER models for the annotated sentences in the same shared latent space (Fig. 2C). In general, the embeddings showed a good separation for the different models, indicating no major overlap of the entities. However, for similar entities, there were some subclusters that served to understand how the models see the different entity terms. For example, ORGANISM clusters into two, one large group containing bona fide organisms, and a smaller one containing cell lines, which were annotated as organisms in the dataset. Similarly, the entity DISEASE formed a subcluster representing cell lines extracted from specific diseased tissues. The larger cluster for DISEASE is composed of diseases of plant, human, and animal origin. For COMPOUND, there were also two clusters, one where the compounds are described in formulaic notation, and another larger one where they are described by the name. The clear clustering into meaningful groups was an indication of the successful grasping of nuances in the entities by the NER models.

We also visualized the embeddings of the RE models on the annotated dataset using UMAP plots. In the representations grouped by the entity (Supplementary Fig. S4), there is a clustering for multi-relation entities (COMPOUND, ORGANISM, and PHENOTYPE). In this case as well, there are subclusters for some types of relations. For example, in ORGANISM, there is a close association of some of the sentence embeddings from INHABITS with SYMBIONT_OF and INHIBITS. As SYMBIONT_OF and INHABITS are overlapping entities, this was expected; however, the INHIBITS relation type was surprising and may refer to the unclear distinction in many cases between positive and negative associations. Supporting this, there was an overlap of some INHABITS and INFECTS relations, which may refer to ambiguous descriptions in the literature. For the same entity, there is a more heterogeneous clustering in the RE models compared to the NER models, which suggests the model learns different sentence structures that result in the same prediction. When grouping by the relation, the clustering is similar (Supplementary Fig. S5). In this case, there is a small subcluster in STRAIN-PHENOTYPE:PRESENTS, which contains exclusively descriptions of bacterial colony morphology. In the INHIBITS UMAP plot, it is interesting to see how some sentences from STRAIN-PHENOTYPE that refer in particular to antimicrobial effects cluster with STRAIN-ORGANISM. Overall, this demonstrates the ability of fine-tuned RE models to understand different relations between entities.

#### Predictions reveal phenotype characterization imbalance

From the total 8457 journals considered, 3.83 million articles were processed by the pipeline. After filtering and quality control, this resulted in a total of 676.4 million sentences that were considered in downstream analyses for the STRAIN prediction. Of these, $\sim$4.3 million were found to contain at least a STRAIN entity term in the corpus ($\sim$0.78%), and were fed into the other NER models for prediction. The number of NER predictions for the other entities (measured as the total number of sentences containing both a STRAIN and such entity) differed slightly from the number of initial annotations (Supplementary Figs S1 and S2). The largest change corresponded to MEDIUM, which climbed two places to reach fifth in the prediction. Exceptions included COMPOUND (second most abundant in both cases) and DISEASE (least abundant in annotations and second least abundant in the predictions). This recapitulates the baseline abundance of entities in the literature and reflects biases in the manual annotation, which resulted in enrichment for specific entities in the training dataset due to ease of labeling and close association with STRAIN entities. These included a large number of novel entity terms not found in the test dataset. After matching STRAIN to reference StrainSelect database and grouping the other entities by similarity, the median number of unique terms was $\sim$141 000 entities (19 000 [SPECIES] to 145 000 [STRAIN]).

### Phenotype information discovery using networks

#### Interconnectivity of network

The annotation and prediction pipelines revealed a rich landscape of microbial phenotype information within the PMC corpus. To study the interconnectivity between all entities and relations, we represented the information as a network where the nodes corresponded to each of the entity terms (belonging to either STRAIN or the other eight entities) and the directed edges to the relations. The result was a large and highly interconnected network where the main component comprised most of the total nodes ($\sim$288 000 versus the total $\sim$308 800 [93.3%]) and edges ($\sim$690 400 versus $\sim$703 000 [98.2%]), suggesting high connectivity among the different entities and relations.

**Figure 3.**
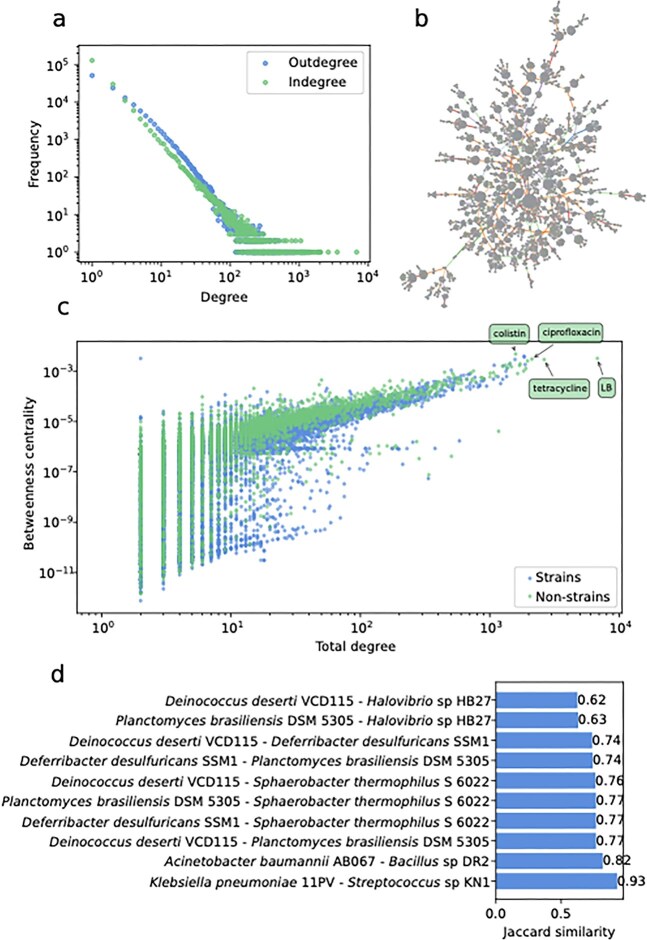
Description of phenotypic network derived from trait annotations in the literature. (**A**) Degree distribution of correlations in the directed phenotype network. On the *x*-axis, the degree is represented, and on the *y*-axis the frequency of these nodes in the network. Note the log scale on both axes. (**B**) Largest connected component obtained after random edge subsampling (10 000 nodes) of the full network. Strain nodes are represented in blue and other entity nodes in green. Different colored directed edges represent different relation types. (**C**) Betweenness centrality of each node against its total degree. In blue are nodes of strain entity and in green nonstrain entity nodes. Labeled are the non-STRAIN nodes found in the top 10 by betweenness centrality. (**D**) Top 10 vertex Jaccard similarity pairs in the network.

The number of incoming and outgoing connections for each node, also known as indegree and outdegree, respectively, showed a characteristic distribution corresponding to a power-law pattern (Fig. 3A). This pattern refers to the logarithmic decrease in degree with frequency of nodes. This means that while most nodes have a single connection, a minority accumulate the greatest number of edges, resulting in the formation of hubs (Fig. 3B). When fitting to a power-law, indegree had a scaling exponent $\alpha$ of 2.1 (KS test against log-normal fit, *P*-value $<$ 0.001) and an outdegree of 2.6 (KS test against log-normal fit, *P*-value $<$ 0.001), which indicates a more even spread of outdegree in the network. Hubs are characterized by a central node with a high degree, connected to many nodes with a single connection. Most of these well-connected hubs corresponded to non-STRAIN entities. However, hubs where the central high-degree node is a strain are also found and correspond to highly characterized strains in the literature. Meanwhile, the largest group of low-degree nodes corresponded to strains or entities that are mentioned only once in the literature.

Power-law distribution of degree occurs in graphs known as scale-free networks. These are often found in nonrandom biological phenomena, such as protein, gene, and metabolic networks [43]. In this case, the structure is likely related to the popularity and influence of certain model strains and pathogens, which accrue the most attention and publications from researchers. This, in turn, results in the hub-pattern exhibited in the network. Similarly, certain properties that are often mentioned when describing strains, such as the Gram stain, create hub patterns with central nodes corresponding to these entities. The scale-free pattern was also evident in the community structure, which, when measured with the Leiden modularity score, resulted in a high modularity ($\sim$0.64), indicating more links within communities than among them [44].

Nodes with the highest outdegree included only bacterial strains (Table 3), mostly belonging to *Escherichia coli* species, corresponding to one of the most popular microbes in research [45]. Additionally, others relevant in, for example, pathogenicity (e.g. *Mycobacterium tuberculosis* H37, a model pathogen) were also highly ranked. The indegree ranking was more varied with regards to entity, owing to the single relation where STRAIN is acting as the target node (COMPOUND-STRAIN:INHIBITS; Table 4). When examining the connectivity of the nodes by comparing their betweenness centrality, the top nodes are again some of the most popular strains together with antibiotics, including tetracycline, colistin, and ciprofloxacin (Fig. 3C). This suggests that these antibiotics, due to their broad spectrum of activity against different strains, may act as the main bridges linking different high-degree hubs together within the network.

**Table 3.** Top 10 nodes by outdegree in the phenotypic network

Node	Outdegree
*Escherichia coli* O157:H7	1398
*Mycobacterium tuberculosis* H37	1350
*Escherichia coli* BL21 DE3	1314
*Escherichia coli* Nissle 1917	1247
*Escherichia coli* DH5	1215
*Lactobacillus* LMG 17291	1208
*Salmonella enterica* ACM 5063	1188
*Pseudomonas putida* KT2440	1142
*Escherichia coli* MG1655	1114
*Pseudomonas aeruginosa* 1C	1102

All nodes belong to the entity STRAIN.

**Table 4.** Top 10 nodes by indegree in the phenotypic network

Node	Indegree	Entity
Mouse	999	ORGANISM
TSB	958	MEDIUM
*Staphylococcus aureus* LMG	703	STRAIN
Plant growth	666	PHENOTYPE
*Escherichia coli* ELI 51	636	STRAIN
PBS	629	MEDIUM
Human	617	ORGANISM
Tetracycline	560	COMPOUND
BHI	559	MEDIUM
Nutrient broth	463	MEDIUM

The Jaccard index, which identifies nodes that are similarly connected to each other within the network, showed pairs of strains that frequently occur together across samples at the highest values rather than close phylogenetic neighbors. Some high Jaccard links connected strains from distant phyla that are found in the same settings, for example *Klebsiella pneumoniae* 11PV with *Streptococcus* sp KN1 and *Acinetobacter baumannii* AB067 with *Bacillus* sp DR2 in clinical or hospital associated contexts, and the concurrent presence of thermophilic or halophilic strains such as *Deinococcus deserti* VCD115, *Sphaerobacter thermophilus* S 6022, *Planctomyces brasiliensis* DSM 5305, and *Rhodothermus marinus* OKD7 in hot or saline environments, consistent with shared habitats rather than close relatedness [46–50]. This showcases the ability of the network to identify relevant ecological or sampling properties shared by strains (Fig. 3D). This also works as an internal control for showing the feasibility in identifying reproducible phenotypic traits for different strains across diverse lineages.

#### Comparison to reference

We used the bacterial phenotype database BacDive [10] to validate the phenotypic network results. This database contains growth conditions alongside qualitative phenotypes that we used to validate our predictions. We identified 9315 strains with annotations in both datasets. We then analyzed some of the most common phenotypes from those by assessing accuracy against conflicting phenotypes and overall overlap. In general, we found high overlap between both manually curated and our predictions (Table 5). However, some annotations including Gram-negative and aerobe phenotype did show relatively lower accuracy (0.510 and 0.762 accuracy, respectively, with *n* = 4656 and *n* = 2206). Using manually curated information could help guide manual annotation for the training of models to address these weak prediction points and improve results in the future. The high number of missing data in the database also highlights the benefits of using predictions to complement such databases.

**Table 5.** Phenotype benchmark using manually annotated phenotype data from BacDive using 9315 strains and common phenotypes

Reference phenotype	Searched phenotype	Accuracy	# Strains	Category
Gram-negative	Gram negative	0.510	4656	PHENOTYPE
Gram-positive	Gram positive	0.762	2206	PHENOTYPE
Anaerobe	Anaerobic	0.830	1604	PHENOTYPE
Spore-forming	Spore	0.833	1548	PHENOTYPE
aAerobe	Aerobic	0.577	3619	PHENOTYPE
Rod-shaped	Rod	0.558	4104	PHENOTYPE
Motile	Motile	0.772	2058	PHENOTYPE
Facultative anaerobe	Facultative anaerob	0.876	1119	PHENOTYPE
Human pathogen	Human pathog	0.965	322	PHENOTYPE
Plant pathogen	Plant pathog	0.984	125	PHENOTYPE
Obligate anaerobe	Obligate anaerob	0.989	99	PHENOTYPE
Coccus	Cocci	0.942	531	PHENOTYPE
Spiral	Spiral	0.997	29	PHENOTYPE
Filamentous	Filament	0.993	50	PHENOTYPE
Thermophilic	Thermophilic	0.950	545	PHENOTYPE
Psychrophilic	Psychrophilic	0.967	303	PHENOTYPE
Halophilic	Halophilic	0.980	55	PHENOTYPE
Pigmented	Pigment	0.987	65	PHENOTYPE
Microaerophile	Microaer	0.929	645	PHENOTYPE
Soil	Soil	0.816	1649	ISOLATE
Marine	Marine	0.899	885	ISOLATE
Clinical	Clinical	0.992	54	ISOLATE
Blood	Blood	0.937	209	ISOLATE
Freshwater	Freshwater	0.974	195	ISOLATE
Thermal	Thermal	0.967	191	ISOLATE
Rhizosphere	Rhizosphere	0.979	137	ISOLATE
Sediment	Sediment	0.918	697	ISOLATE
Wastewater	Wastewater	0.978	179	ISOLATE
Fecal	Fecal	0.948	487	ISOLATE
Marine medium	Marine	0.897	928	MEDIUM
Nutrient agar	Nutrient agar	0.914	703	MEDIUM
Tryptic	Tryptic	0.840	1312	MEDIUM
MRS	mrs	0.955	236	MEDIUM
R2A	r2a	0.931	561	MEDIUM

#### Inferring strain–strain correlations from trophic networks

The understanding of microbe–microbe correlations is very relevant for predicting the assemblage and function of microbial communities. However, these correlations are notably hard to study due to experimental constraints, as their inference traditionally entails pairwise growth testing of different strains in the lab and abundance analyses across different samples to study co-occurrence patterns [51, 52]. Trophic interactions based on intermediary metabolic compounds have been shown to dictate dynamics in such microbial communities and can be used as a method for inferring microbe-microbe correlations to complement more classical approaches [53, 54].

**Figure 4.**
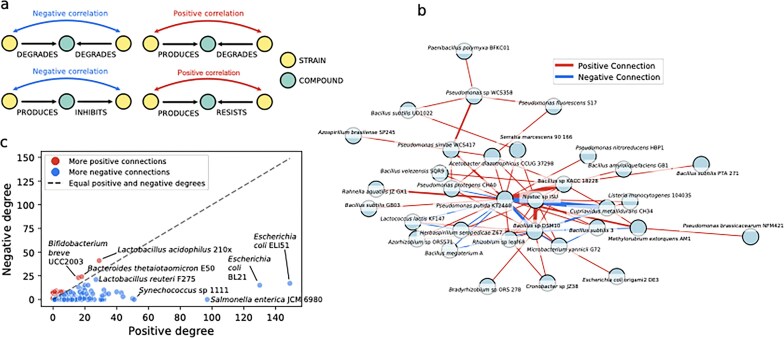
Microbe–microbe correlation inference. (**A**) Triad combinations in the phenotypic network used to infer trophic microbe–microbe interactions. (**B**) Inferred strain–strain correlations for bacteria inhabiting *A. thaliana* plants. The width of the edges represents the weight of the connection. (**C**) Node in and out-degree distribution for strain–strain correlations in the inferred trophic interaction network relating to human-colonizing bacteria.

We used the knowledge network of phenotypes to infer strain-strain correlations by constructing trophic networks based on the LLM predictions from the literature. We built these by grouping strains that inhabited the same hosts or environments according to the INHABITS relation. We next performed an analysis of triads (three contiguously connected nodes) in this subnetwork to infer four types of common trophic connections (Fig. 4A; see “Community interaction”). They included two negative interactions related to competition and inhibition and two positive interactions related to cross-feeding and resistance.

In the constructed strain–strain interaction networks, some nodes were highly connected. For example, when looking at strains associated with *Arabidopsis thaliana* plants (*n* = 91), these included strains such as *Pseudomonas putida*, which had many positive correlations both in number and weight (Fig. 4B). As a common goods producer, *P. putida* has been studied in the context of acting as a driver of microbiome diversity in the rhizosphere of plants [55]. This shows that these inferred trophic correlations, although biased by the information found in the literature, nevertheless reflect actual biological processes. As found previously, positive connections were more prevalent than negative ones in *A. thaliana*-associated communities, suggesting that community assembly and stability are driven more by cooperation than by competition [56].

When looking at other environments, such as those associated with humans, which contained a higher number of nodes (*n* = 1436), we found strains that were highly interconnected for both positive and negative links to other strains (Fig. 4C). Commensal bacteria such as *E. coli* and *Bacillus*, which are generally expected to have a neutral effect on the host, were the highest when ranking positive correlations. Meanwhile, probiotic bacteria such as *Lactobacillus* and *Bifidobacterium* had a higher ratio of negative to positive connections. This relates to the capacity of probiotics in controlling the microbial community by inhibiting pathogenic competitors [57]. Overall, while our analysis does not capture the full ecological complexity of interactions, it presents an example of applying the generated phenotypic network to derive biological insights.

### Phenotype–gene correlations identify key proteins

#### Ranking gene importance

In order to find correlations between the genomes of bacteria and their phenotype as predicted by the network, we used gradient boosting. Linking genomes and the phenotype network allowed us to pinpoint specific microbial genes across several taxa as responsible for particular phenotypes. In total, we found 3455 entity–entity significant correlations containing a STRAIN, mainly corresponding to relations STRAIN-EFFECT:PRESENTS (n = 808), STRAIN-MEDIUM:GROWS_ON (*n* = 731) and STRAIN-COMPOUND:RESISTS (*n* = 406). This observation partially parallels the relation annotations, further suggesting that increasing the size of the training dataset could lead to a larger number of correlations (Fig. 1C). The enriched GO terms for the relevant genes in each of the categories overlapped greatly (Supplementary Fig. S6). Membrane- and catalytic-activity-related terms were common to most of the relations, highlighting the relevance of proteins that fit these categories in the adaptation and evolution within different environments, eventually resulting in different phenotypes [58, 59]. Interestingly, two of the INHIBITS relations were enriched in biosynthetic terms, showing the importance of the production of antimicrobials in such negative correlations [60].

#### Phenotypic traits

The highest importance annotations belonged mostly to STRAIN-MEDIUM:GROWS_ON and STRAIN-DISEASE:ASSOCIATED_WITH relations, but they were varied and did not correlate to the higher abundance entity–entity relation (Table 6). Additionally, these did not include entity terms that showed the highest degree, indicating that more connections to strains did not necessarily lead to a better significance of correlations. Among the highest importance annotations are known direct correlations between phenotype and functional annotations. For example, many probiotic bacteria are enriched in secreted peptidases, including C69 type, which participate in the breakdown of proteins that improve digestion and feed a healthy gut microbiome [61]. Other annotations with known correlations in literature include several LB-associated features under STRAIN-MEDIUM:GROWS_ON (pili assembly chaperone/PapC-like usher, HAMP signaling modules, and enterobactin utilization via the Fes esterase) that are coherent with adhesion, chemotaxis, and iron acquisition on rich media [62–66].

**Table 6.** Annotations with top 35 importance scores for their correlation with phenotype. Importance ranking represents the ranking per category of the particular annotation

Relation	Term	Gene description	Imp.	Acc.	Rank
STRAIN-MEDIUM:GROWS_ON	LB	Pili assembly chaperone, N-terminal	145.38	0.91	1
STRAIN-DISEASE:ASSOCIATED_WITH	Cholera	Hemolysin, beta-prism lectin	127.61	0.98	1
STRAIN-MEDIUM:GROWS_ON	LB	Amino acid permease/ SLC12A domain	110.23	0.91	2
STRAIN-MEDIUM:GROWS_ON	MRS	Exoribonuclease, phosphorolytic domain 1	101.90	0.98	1
STRAIN-MEDIUM:GROWS_ON	LB	PapC-like, C-terminal domain	101.75	0.91	3
STRAIN-DISEASE:ASSOCIATED_WITH	HUS	Group 4 capsule polysaccharide formation lipoprotein GfcB	72.00	0.96	1
STRAIN-SPECIES:INHIBITS	*E. coli*	Uncharacterised protein YjbT	71.62	0.80	1
STRAIN-ISOLATE:INHABITS	Human stool	Haem-binding HasA	71.33	0.99	1
STRAIN-DISEASE:ASSOCIATED_WITH	Cholera	Flagellin, N-terminal domain	67.20	0.98	2
STRAIN-COMPOUND:RESISTS	Colistin	Protein of unknown function DUF406	66.81	0.97	1
STRAIN-DISEASE:ASSOCIATED_WITH	Melioidosis	Protein of unknown function DUF3564	65.00	1.00	1
STRAIN-MEDIUM:GROWS_ON	MRS broth	Exoribonuclease, phosphorolytic domain 1	60.96	0.99	1
STRAIN-SPECIES:INHIBITS	*Campylobacter jejuni*	Protein of unknown function DUF2972	55.84	0.99	1
COMPOUND-STRAIN:INHIBITS	Erythromycin	CRISPR-associated endonuclease Cas9, wedge domain	55.70	0.98	1
COMPOUND-STRAIN:INHIBITS	Colistin	Uncharacterised protein family, YcgJ	55.63	0.96	1
STRAIN-ORGANISM:INFECTS	Mice	M protein trans-acting positive regulator (MGA), PRD domain	51.99	0.80	1
STRAIN-DISEASE:ASSOCIATED_WITH	Gastroenteritis	Protein of unknown function DUF3373	51.93	0.97	1
STRAIN-EFFECT:PRESENTS	Fermentative capacity	Interleukin-1 receptor-associated kinase1-binding protein 1/DUF541	48.56	1.00	1
STRAIN-COMPOUND:RESISTS	Vancomycin	Protein of unknown function DUF961	48.48	0.98	1
STRAIN-ORGANISM:INFECTS	Human	Peptidoglycan O-acetylesterase, N-terminal	48.45	0.95	1
STRAIN-DISEASE:ASSOCIATED_WITH	ipd	DpnD, N-terminal domain	47.78	0.98	1
STRAIN-ORGANISM:INFECTS	BALB/c mice	Peptidase M17, leucyl aminopeptidase, C-terminal	47.33	0.98	1
STRAIN-COMPOUND:RESISTS	Colistin	YdbH-like	45.21	0.97	2
STRAIN-DISEASE:ASSOCIATED_WITH	hus	Protein of unknown function DUF1627	44.92	0.96	2
STRAIN-MEDIUM:GROWS_ON	TSB	Phenol-soluble modulin beta protein	43.93	0.97	1
STRAIN-ORGANISM:INFECTS	Mice	Bacteriocin, class IIb, lactacin-related	43.67	0.80	2
STRAIN-MEDIUM:GROWS_ON	Luria–Bertani	NanQ anomerase/TabA/YiaL family	43.31	0.97	1
STRAIN-MEDIUM:GROWS_ON	LB	Enterochelin esterase, N-terminal	42.91	0.91	4
STRAIN-COMPOUND:RESISTS	Fluoroquinolones	Protein of unknown function DUF2972	42.65	0.98	1
STRAIN-PHENOTYPE:PRESENTS	Probiotic	Peptidase C69	42.20	0.95	1
STRAIN-DISEASE:ASSOCIATED_WITH	Cholera	(Na+)-NQR maturation factor NqrM	41.95	0.98	3
STRAIN-PHENOTYPE:PRESENTS	Nitrogen fixing	Protein of unknown function DUF1236	41.94	0.99	1
STRAIN-MEDIUM:GROWS_ON	LB	HAMP domain	41.59	0.91	5
STRAIN-SPECIES:INHIBITS	*S. aureus*	MrpA C-terminal/MbhD	40.98	0.84	1
STRAIN-MEDIUM:GROWS_ON	Luria–Bertani	YehS-like	40.53	0.97	2

Direct disease-linked signals among the top items also align with established mechanisms. For cholera, hemolysin bearing a C-terminal β-prism lectin domain, flagellin (N-terminal), and the Na$^+$-NQR maturation factor (NqrM) are consistent with *Vibrio cholerae* cytolysis, motility-dependent colonization, and sodium-coupled respiration during infection [67–70]. For infection models, STRAIN-ORGANISM:INFECTS entries include the Mga PRD-domain regulator in *Streptococcus* (a well-characterized virulence regulator in mice) and peptidoglycan O-acetyl/de-O-acetyl systems linked to lysozyme evasion during human infection [71–73].

Several high-ranking pairs are indirectly supported or appear biologically plausible but under-documented. The hemophore HasA paired with the isolation source “human stool” fits widespread heme scavenging among enteric bacteria [74] and coupling of erythromycin with a Cas9 (wedge) domain under COMPOUND-STRAIN:INHIBITS agrees with CRISPR–Cas limiting acquisition of resistance elements [75–77].

Our analysis revealed genes with high importance across multiple relationship types, demonstrating biologically coherent patterns (Table 7). For instance, genes associated with antibiotic resistance mechanisms showed convergent evidence: the highest-ranked gene, protein of unknown function DUF406, exhibited high importance for both colistin resistance and antibiotic resistance phenotype presentation, consistent with known LPS modification mechanisms underlying colistin resistance [78]. Similarly, D,L-carboxypeptidase showed high importance for both inhibiting *Campylobacter jejuni* and presenting microaerophilic phenotype, reflecting the established role of peptidoglycan-modifying carboxypeptidases (Pgp1/Pgp2) in maintaining the helical morphology and microaerophilic lifestyle of this pathogen [79]. Capsule biosynthesis genes demonstrated coupled importance for association with hemolytic uremic syndrome and resistance to $\beta$-lactam antibiotics, linking virulence and antimicrobial resistance traits. The ability of independent relationship types to converge on the same gene annotations with related biological functions demonstrates the robustness of our method and its capacity to retrieve biologically sound information related to complex phenotypic traits.

**Table 7.** Genes with high importance across multiple relationship types

Gene	Imp.	Key relationships
Protein of unknown function DUF406	66.8	COMPOUND:RESISTS: colistin (67)
		EFFECT:PRESENTS: antibiotic resistance (6)
Interleukin-1 receptor-associated kinase1-binding protein 1	48.6	EFFECT:PRESENTS: fermentative capacity (49)
		COMPOUND:RESISTS: erythromycin (6)
Surface composition regulator	40.3	COMPOUND-STRAIN:INHIBITS: fosfomycin (40)
		EFFECT:PRESENTS: antibiotic resistance (15)
Collagen-binding domain	37.1	ORGANISM:INFECTS: humans (37)
		PHENOTYPE:PRESENTS: facultative anaerobic (12)
SH3b2-type SH3 domain	32.9	ORGANISM:INFECTS: humans (33)
		DISEASE:ASSOCIATED_WITH: gastroenteritis (21)
Domain of unknown function DUF4261	29.7	DISEASE:ASSOCIATED_WITH: gastroenteritis (30)
		ORGANISM:INFECTS: chicken (6)
Protein of unknown function DUF819	29.4	ORGANISM:INHABITS: bovine (29)
		EFFECT:PRESENTS: surface (7)
D,L-carboxypeptidase, peptidase domain	28.9	SPECIES:INHIBITS: *Campylobacter jejuni* (29)
		PHENOTYPE:PRESENTS: microaerophilic (8)
Capsule biosynthesis GfcC-like, C-terminal	28.2	DISEASE:ASSOCIATED_WITH: hemolytic uremic syndrome (28)
		COMPOUND:RESISTS: piperacillin/tazobactam (5)
YetF, C-terminal domain	26.4	PHENOTYPE:PRESENTS: gram negative (26)
		COMPOUND:RESISTS: colistin (6)

#### Host association and pathogenicity

To effectively study genes associated with host colonization and infection, we examined their patterns in our correlation study. Our approach allows us to identify genes that may be relevant for adapting to specific hosts. For the relation STRAIN-ORGANISM:INHABITS, we found a significant phylogenetically diverse set of organism hosts, including animals and plants (Supplementary Fig. S7). The lack of overlap in high-importance genes among hosts likely relates to the different strategies that bacteria use to adapt to a particular host and to the large phylogenetic diversity of hosts in the dataset. Plant-associated hosts prominently feature necrosis-inducing proteins (NLPs), cellulose-binding carbohydrate-binding module family 3 (CBM3), and pectate lyases—consistent with adhesion to and remodeling of plant cell walls and with NLPs acting as MAMPs in dicots [80–82]. Additional recurrent entries include stress/competence and signaling components such as RecR (zinc finger; RecFOR pathway), Smf/DprA–SLOG (natural transformation), and histidine kinase/HSP90-like ATPase domains (two-component signaling) [83–85].

Performing a similar approach on the ORGANISM:INFECTS relation, we also noticed a lack of overlap (Supplementary Fig. S8). However, when looking at the enriched annotations, we found these more directly relating to pathogenicity and not necessarily symbiotic or neutral effects on the host, suggesting that the models are able to capture the difference. Prominent features include a class IIb (two-peptide) bacteriocin (lactacin-related), collagen-binding domains, the IgG-degrading protease IdeS, peptidoglycan O-acetylesterases, SH3b cell-wall–binding domains, and Type VI secretion system components—specifically a phospholipase effector (Tle1-like) together with the Tla3 adapter—each mapping to established mechanisms of interbacterial antagonism, adhesion/immune evasion, lysozyme resistance/cell-wall remodeling, and T6SS-mediated competition [86–92]. Additional items visible include transcriptional/stress regulators (MarR-type HTH), ribosome-associated maturation elements (RimP C-terminus; Trm112-like; 23S rRNA methylase leader peptide), and toxin–antitoxin/defense modules (HEPN/AbiU2-like, AbiEii nucleotidyltransferase, HipA-like, HigB-like), which are frequently implicated in persistence, translation control, and phage/competitor interactions during infection [93–96].

Other virulence- and colonization-associated features present in our figures include the M protein *trans*-acting positive regulator (MGA/PRD domain), penicillin-binding protein A (dimerization domain), and ABC transporter components (type 1, GsiC-like; ABC3 permease), consistent with adhesion/regulatory control, envelope remodeling, and glutathione import linked to oxidative-stress defense [97–99]. Given the prevalence of these, we hypothesize that the other enzymes that do not directly fit the virulence paradigm may represent potential virulence factors indirectly advancing pathogenicity in a particular host. These include multiple entries labeled as toxins/antitoxins or mobile elements (e.g., HEPN/AbiU2-like, AbiEii, tail-sheath, and other phage proteins), supporting roles in interbacterial antagonism and horizontal gene flow within hosts [96].

Antimicrobial compound production and defense also feature heavily in the subset of high-importance genes related to host colonization in both relations. In particular, Supplementary Fig. S8 highlights a class IIb bacteriocin (lactacin-related), aligning with interbacterial competition during infection [86]. Resistance/evasion–linked terms present in the figures include MarR-type HTH regulators, peptidoglycan *O*-acetylesterases, SH3b cell-wall–binding domains (in peptidoglycan hydrolases), penicillin-binding protein A domains, and ABC transporters (GsiC-like; ABC3 permease) [89, 90, 93, 98, 99]. The prevalence of terms belonging to this category in both host-associated relations highlights the current problem of antibiotic resistance development in fighting infection. Finally, numerous domains of unknown function (DUFs) entries appear among the top features in both figures, underscoring opportunities for discovery and the dependence of our approach on accurate functional annotation.

#### Positive selection in highly important features

**Figure 5.**
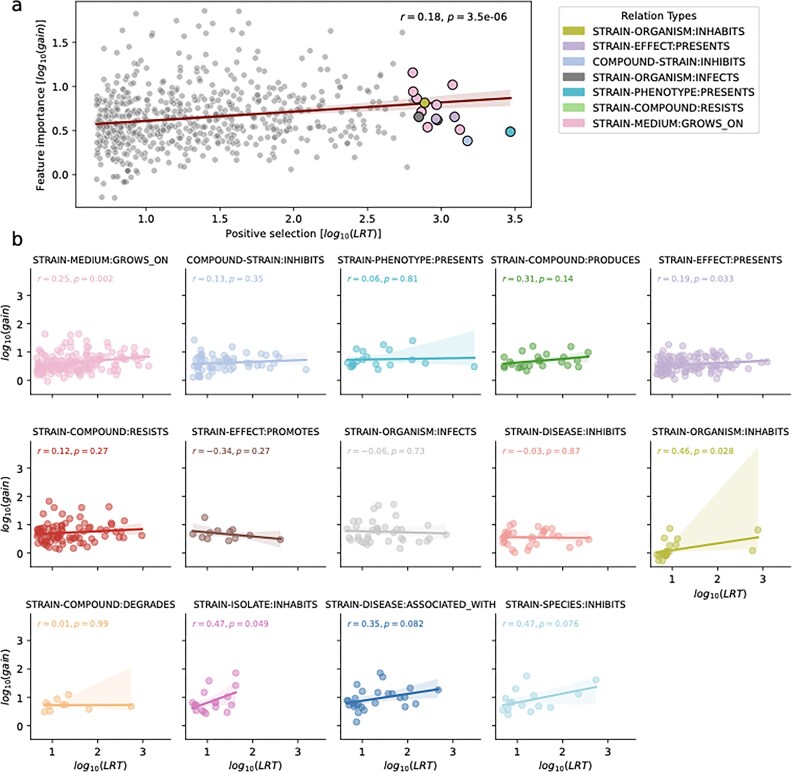
Positive selection in relevant genes. (**A**) Positive pressure correlation among the top importance annotations and the LRT for all significant protein families with evidence of positive selection. Top 15 correlations by $\log _{10}(\text{LRT})$ are highlighted with a color representing their relation. (**B**) Positive pressure correlation among the top importance annotations and the LRT for positive selection of the highest populated relation containing at least 5 gene correlations.

When examining these correlations and their highest importance genes, we observed an enrichment in genes under positive selective pressures. Of all the genes tested (2466), 646 (26.2%) showed evidence for positive selection. This is much higher than the predicted base rate for genes under detectable selection in a genome, which generally does not exceed 20% [100, 101]. The prevalence of positive selection underscores the relevance of these highly correlated genes to particular phenotypes in adaptation. Overall, there was a small positive correlation between the phenotype importance values and the evidence for positive selection as measured with the LRT (Fig. 5A and Table 8).

**Table 8.** Gene–phenotype correlations with high evidence of positive selection

Gene description	Term	Relation	logLRT	logImp.
TonB-dependent receptor-like, β-barrel	Denitrifying	STRAIN-PHENOTYPE:PRESENTS	3.47	0.49
AMP-binding enzyme, C-terminal domain	DMSO	COMPOUND-STRAIN:INHIBITS	3.18	0.38
Condensation domain	YMA	STRAIN-MEDIUM:GROWS_ON	3.13	0.51
CAAX prenyl protease 2/Lysostaphin resistance protein A-like domain	PBS	STRAIN-MEDIUM:GROWS_ON	3.08	1.02
YSIRK Gram-positive signal peptide	Daptomycin	STRAIN-COMPOUND:RESISTS	2.97	0.62
Rieske _2Fe-2S_ iron-sulphur domain	FBS	STRAIN-MEDIUM:GROWS_ON	2.97	0.79
Peptidase, C-terminal, archaeal/bacterial	Lipid productivity	STRAIN-EFFECT:PRESENTS	2.96	0.63
Acyl transferase domain	7H10 agar	STRAIN-MEDIUM:GROWS_ON	2.91	0.54
AMP-binding enzyme, C-terminal domain	*Fusarium*	STRAIN-ORGANISM:INHABITS	2.89	0.81
ABC transporter periplasmic binding domain	IMDM	STRAIN-MEDIUM:GROWS_ON	2.87	0.71
Autotransporter β-domain	Swine	STRAIN-ORGANISM:INFECTS	2.85	0.66
LacI-type HTH domain	M17 broth	STRAIN-MEDIUM:GROWS_ON	2.83	0.86
Acyl transferase domain	Sabouraud dextrose agar	STRAIN-MEDIUM:GROWS_ON	2.81	0.94
Mucin-binding domain	MRS agar	STRAIN-MEDIUM:GROWS_ON	2.81	1.16
Polyketide synthase dimerization element domain	ISP4 agar	STRAIN-MEDIUM:GROWS_ON	2.80	0.84
MarR-type HTH domain	DA1116	STRAIN-ORGANISM:INHABITS	2.77	0.08
Aminotransferase class V domain	Lignocellulose	STRAIN-COMPOUND:DEGRADES	2.74	0.68
MrpA C-terminal/MbhD	*S. aureus*	STRAIN-SPECIES:INHIBITS	2.74	1.61
Cation-transporting P-type ATPase, N-terminal	Stress tolerance	STRAIN-EFFECT:PRESENTS	2.69	0.87
CBS domain	Probiotic properties	STRAIN-EFFECT:PRESENTS	2.68	0.41
6-Hydroxymethylpterin diphosphokinase MptE-like	Diarrheal disease	STRAIN-DISEASE:ASSOCIATED_WITH	2.67	1.26
RHS protein	Mueller–Hinton	STRAIN-MEDIUM:GROWS_ON	2.62	0.74
RTX calcium-binding nonapeptide repeat	Number	STRAIN-EFFECT:PROMOTES	2.61	0.48
Mub B2-like domain	Bile salts	STRAIN-COMPOUND:RESISTS	2.59	1.14
Topoisomerase C-terminal repeat	RPMI 1640	STRAIN-MEDIUM:GROWS_ON	2.59	1.12
Porin, opacity type	Cefixime	COMPOUND-STRAIN:INHIBITS	2.58	0.83
S-layer protein, C-terminal domain, *Lactobacillus*	AD	STRAIN-DISEASE:INHIBITS	2.58	0.49
NB-ARC	Itraconazole	STRAIN-COMPOUND:RESISTS	2.57	0.54
Transglutaminase-like	PYG	STRAIN-MEDIUM:GROWS_ON	2.57	0.76

The logLRT and logImp. columns represent the log-LRT and the log importance values, respectively. Entries with logLRT > 2.5 for each relation are depicted.

Examples of genes under strong positive selection include a TonB-dependent receptor–like $\beta$-barrel associated with denitrifying conditions, consistent with TonB-dependent transport mediating acquisition of scarce substrates [102]; YSIRK-motif signal peptides that target secreted proteins to septal membranes in Gram-positive bacteria [103, 104]; and autotransporter $\beta$-domains linked to host infection [105, 106]. We also observe ABC-transporter periplasmic-binding domains among the top hits, aligning with the central role of ABC systems in nutrient uptake at host–pathogen interfaces [107, 108], and phosphopantetheine-binding acyl carrier protein (ACP) domains among psychrophile-associated terms, consistent with membrane lipid remodeling as a hallmark of cold adaptation [109–111].

We grouped these correlations and found that for the most populated relations, there was a significant positive correlation to the highest importance genes, including STRAIN-MEDIUM:GROWS_ON, STRAIN-EFFECT:PRESENTS and STRAIN-PHENOTYPE:PRESENTS. This suggests that diversifying selection is driving adaptation behind particular phenotypes, including adaptation to media and metabolite production. In the other relations, although mostly showing a positive trend, there was no significant pattern of correlation (Fig. 5B). This lack of significant positive correlation could be explained by the bias in prediction and the differences in the distribution of relation types tested for positive selection.

### Limitations

While our NER and RE approach is able to capture strain-entity relationships on data where the strain is named, it lacks the ability to do so when the strain is not explicitly repeated. The latter includes large-scale studies where only high-level patterns are reported or in instances where the strain has been named previously. We have partially mitigated the latter problem by including entire paragraphs instead of sentences when these were below 512 characters. Although the training data did not contain cross-sentence relations, we did find examples for this in the predictions after manual curation. To improve on this approach, a strategy of co-reference resolution could be implemented [112]. This method is based on replacing the full strain names anywhere they are referenced in an article. Another limitation in our NER/RE approach includes the filtering for articles in English, which could be addressed by pre-translating the corpus or using multilingual models [113].

Regarding the phenotypic network structure, the pattern of hubs highlights the need to develop methods to extract information that capitalize on this imbalance in phenotype descriptions. Given this structure, future work may consider using phylogenetic relationships to highlight taxonomy-specific correlations and infer patterns in closely related strains. Moving forward, improvements could also be made to the network analysis by weighting confidence by frequency of independently reported traits.

While our approach is less reliable for creating accurate annotations and more sensitive to publication bias than manual curation, it has the potential to scale better and become more complete as the corpus grows and computational capacity improves. Additionally, our approach is restricted to qualitative data and lacks the nuances of quantitative measures, such as optimal growth temperature or pH. These limitations may also be viewed as advantages, as they allow experimental researchers to easily obtain a snapshot of the phenotype information of their strain of interest as it appears in the literary corpus. This can be highly advantageous for sensibly directing research resources to solve specific problems.

## Conclusion

This work demonstrates the potential of NLP to unlock rich bacterial phenotype information from scientific literature. The constructed phenotype network revealed a power-law distribution with hubs consisting of well-studied strains and common phenotypes, highlighting the ability of the network to identify reproducible traits as well as potential biases in research focus. This emphasizes the importance of conducting more research on less-studied and exotic strains, allowing future predictions to be more generalizable. Nonetheless, the network enabled trophic network inference from strains associated with various environments to assess microbe–microbe interactions. The gradient boosting analysis successfully inferred phenotype–gene correlations, agreeing with the current scientific literature and uncovering novel patterns related to traits, pathogenicity, and host association. Notably, we found an enrichment in antimicrobial production and defense proteins for host association and pathogenicity. Many of these novel findings could be useful for experimental researchers, as they allow potential candidate genes to be identified when investigating certain phenotypes, presenting a valuable resource for the scientific community. Importantly, instances of positive selection highlighted genes underlying adaptive phenotypes in the phenotypic relations.

In summary, this work paves the way for large-scale, automated exploration of the relationships between microbial genotype and phenotype and provides examples and strategies to analyze such extensive data. Looking ahead, improvements that tackle current constraints, namely the method’s lack of bacterial phylogeny awareness and the limited handling of cross-sentence references, will be essential to broaden the approach’s accuracy and scope.

## Supplementary Material

lqaf174_Supplemental_File

## Data Availability

The full pipeline to reproduce all analyses and train all models in this manuscript is found in https://github.com/danielzmbp/NLP4Pheno. The full pipeline to reproduce all analyses and train all models in this manuscript is found in https://github.com/danielzmbp/NLP4Pheno. The code to reproduce all analyses is available at https://doi.org/10.5281/zenodo.17473327. The complete dataset including NER predictions, relation extraction results, and XGBoost phenotype predictions is available at https://doi.org/10.5281/zenodo.17474463.

## Notes

Present address: Earlham Institute, Norwich, United Kingdom
