# Supplementary material for: Integrating natural language processing and genome analysis enables accurate bacterial phenotype prediction
Source: NAR Genom Bioinform. 2025 Dec 29;7(4):lqaf174. doi: 10.1093/nargab/lqaf174 (PMC12746109; doi:10.1093/nargab/lqaf174)
Supplement: lqaf174_Supplemental_File [file lqaf174_supplemental_file.pdf]

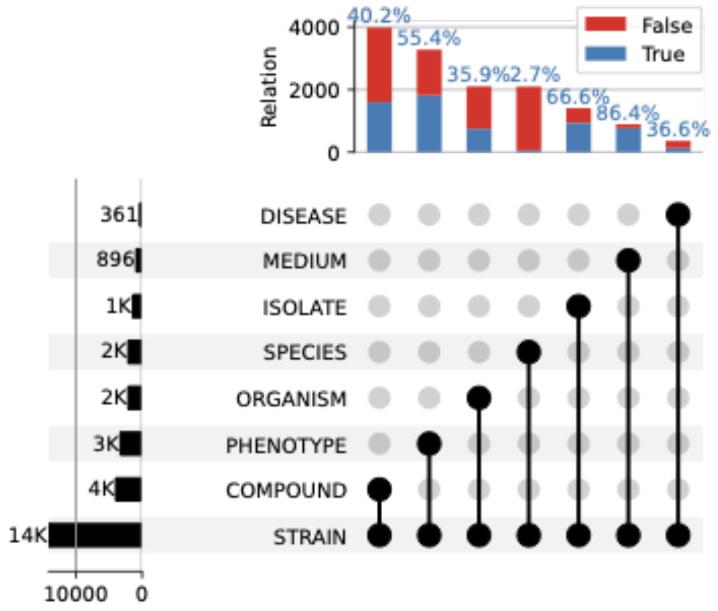

Supp. Fig. 1. Upset group plot showing the sentence co-occurrence of `STRAIN` entity terms with others in the annotated dataset. Labeled in blue on top of each bar are the percentage of entities with a relation extraction (RE) annotation among them.

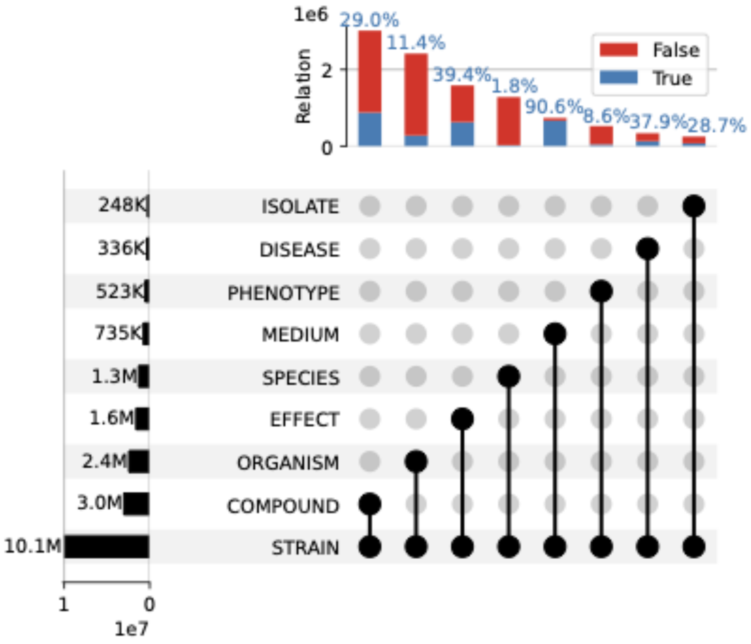

Supp. Fig. 2. Overview of predictions in the PubMed corpus. Upset group plot showing the sentence co-occurrence of `STRAIN` entity terms with others in the predicted dataset. Labeled in blue on top of each bar is the percentage of entities with a relation extraction (RE) annotation among them.

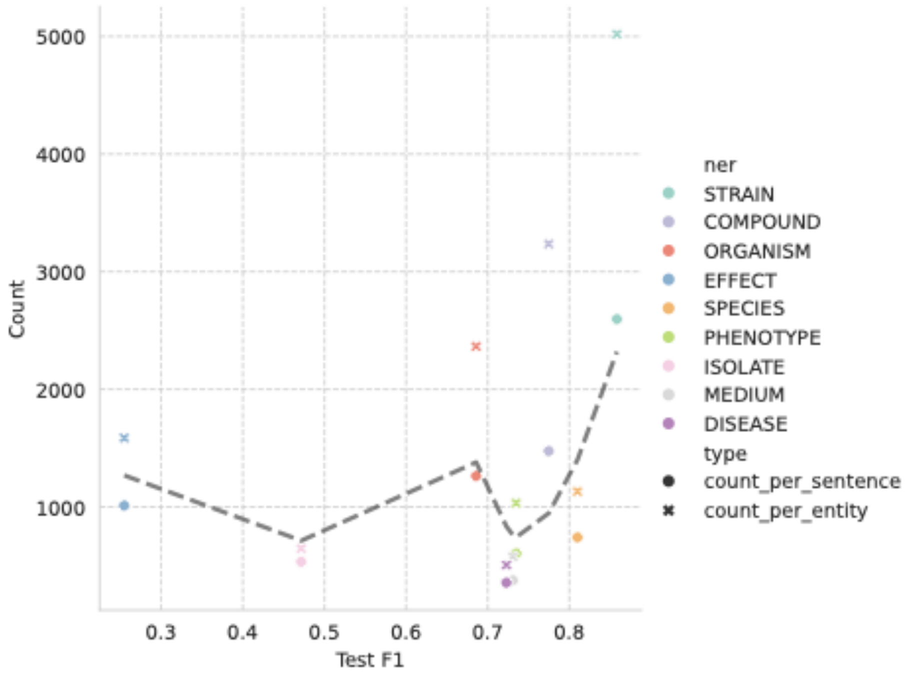

Supp. Fig. 3. Correlation of the number of labeled entities in the annotation dataset to model performance. The counts by either sentence (circle) or entity (cross) are displayed correlated to the strict F1 performance on the test set after training, grouped by the colored entity. Trend line is drawn using LOESS smoothing over all points.

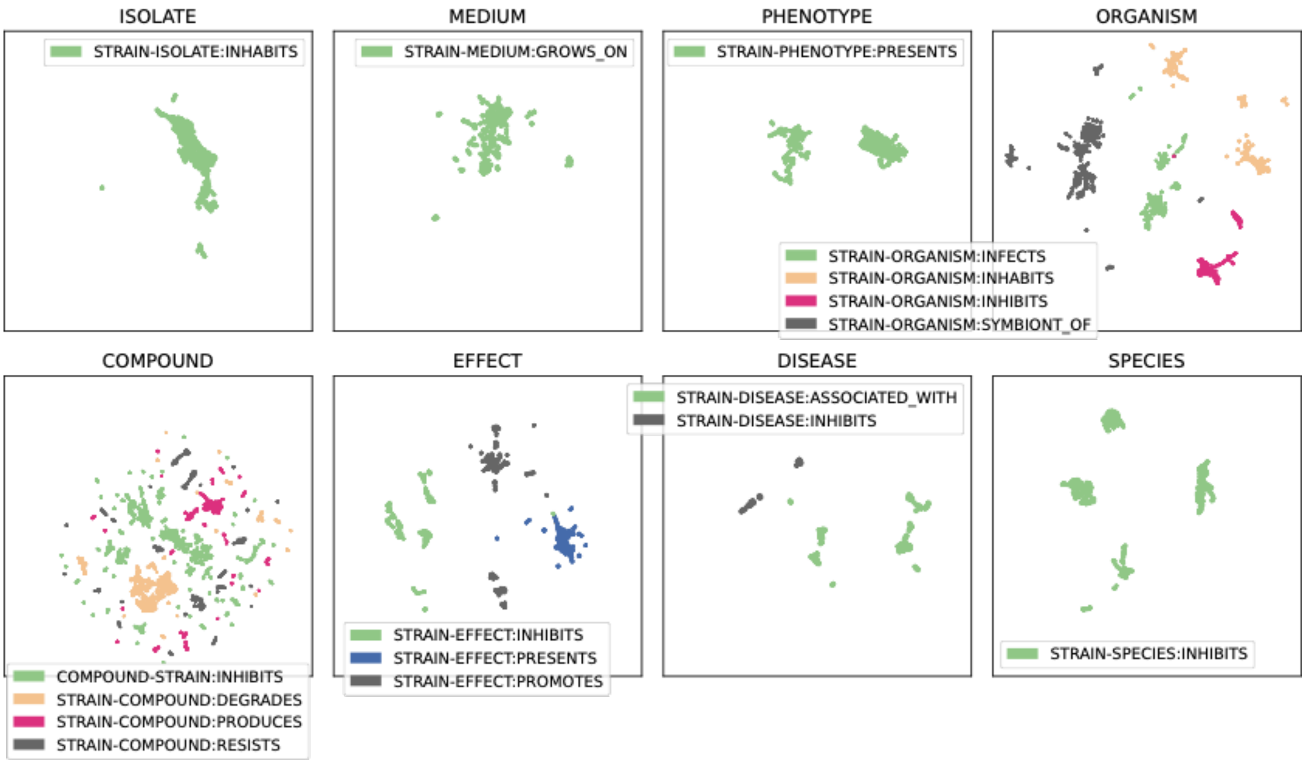

Supp. Fig. 4. UMAP representation of sentence embeddings in the training set for each of the different relation extraction models, grouped by entity. Displayed are only sentences that are positive for the presence of that particular relation. Axes correspond to UMAP dimensions 1 and 2.

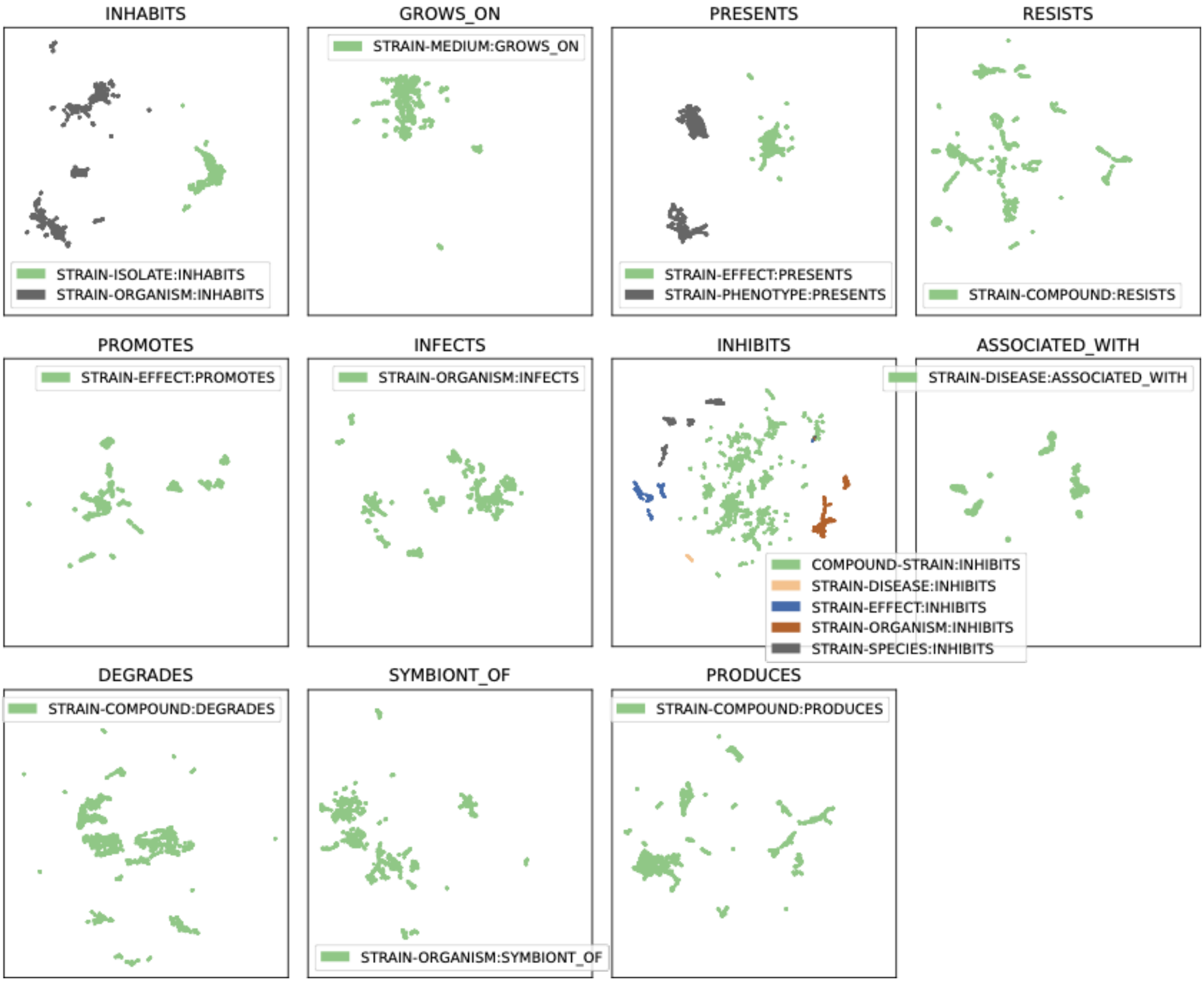

Supp. Fig. 5. UMAP representation of sentence embeddings in the training set for each of the different relation extraction models, grouped by relation. Displayed are only sentences that are positive for the presence of that particular relation. Axes correspond to UMAP dimensions 1 and 2.

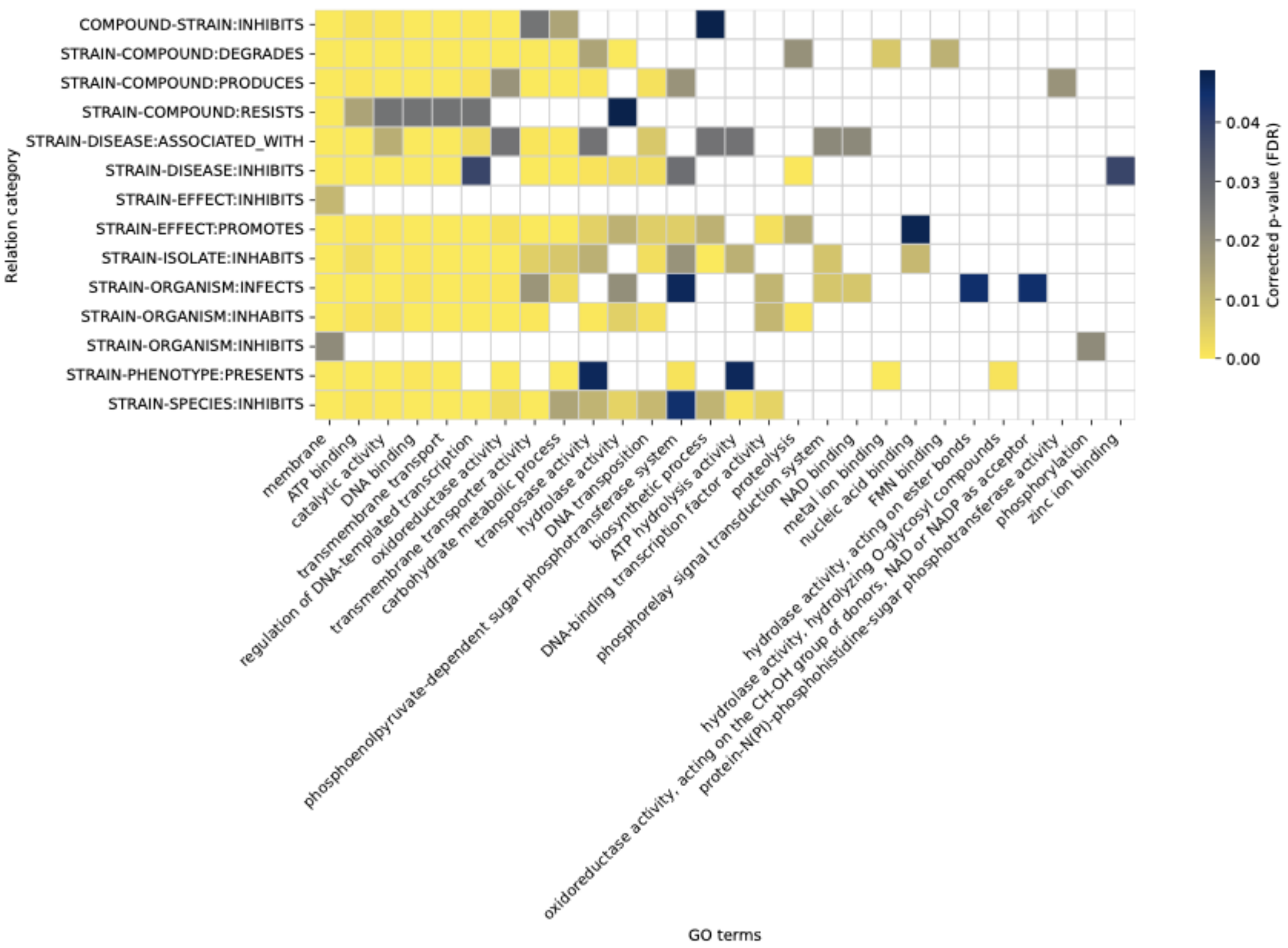

Supp. Fig. 6. GO enrichment of high-importance genes in correlation to phenotype, grouped by their relation category.

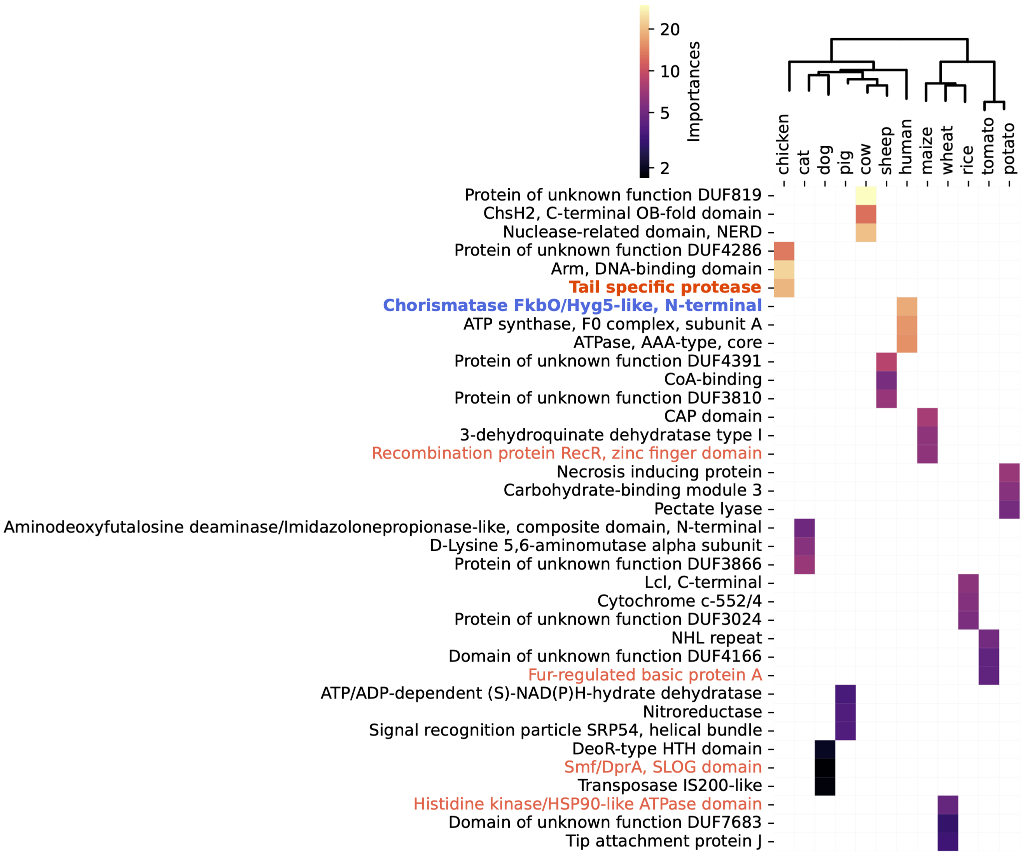

Supp. Fig. 7. Prevalence of bacterial genes in the STRAIN-ORGANISM:INHABITS ORGANISM relations. At the top, the phylogenetic tree represents the distance between the hosts. Highlighted genes in red correspond to antimicrobial resistance and in blue to antimicrobial production and activity. Opacity reflects literature support.

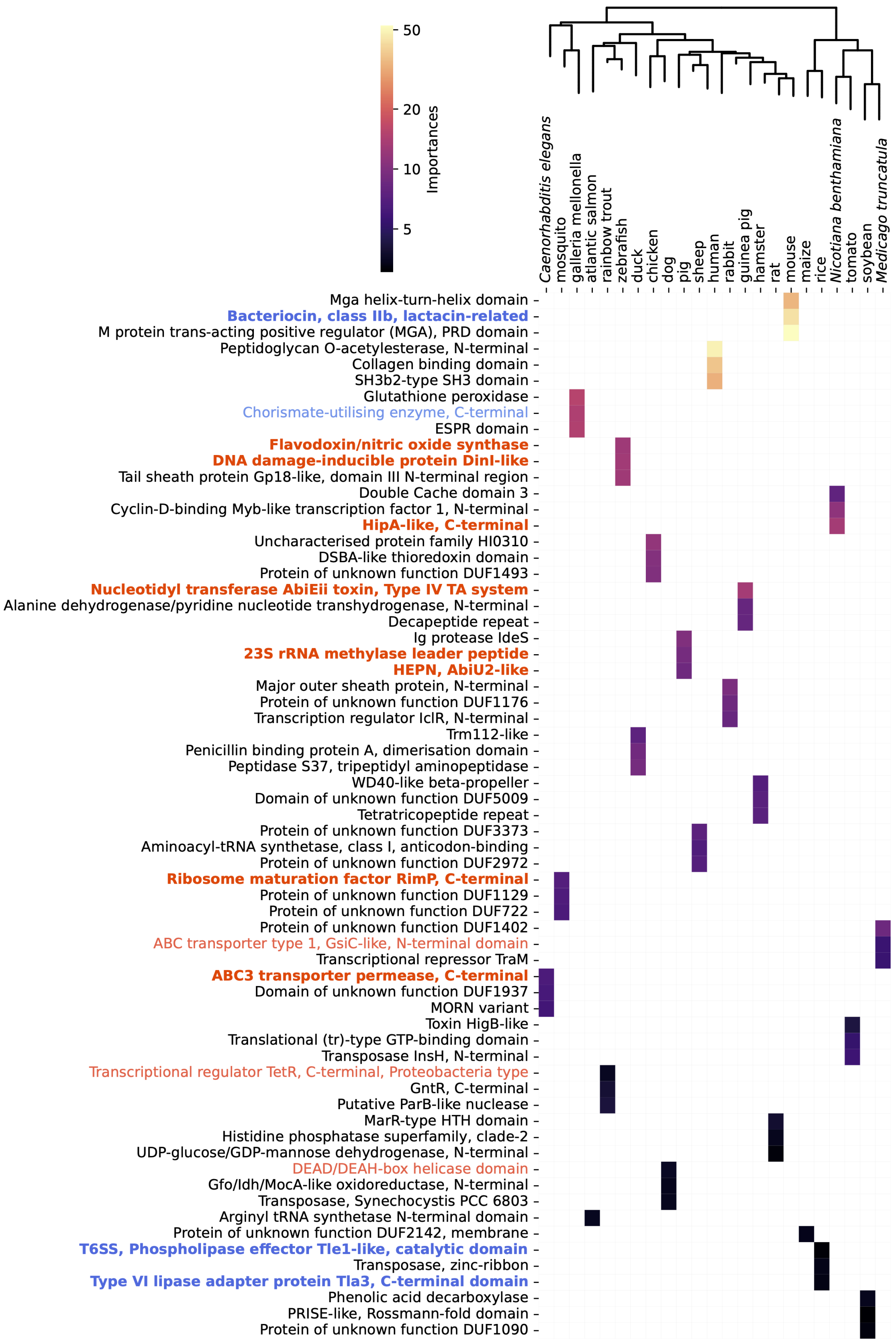

Supp. Fig. 8. Prevalence of bacterial genes in the STRAIN-ORGANISM:INFECTS relations. At the top, the phylogenetic tree represents the distance between the hosts. Highlighted genes in red correspond to antimicrobial resistance and in blue to antimicrobial production and activity. Opacity reflects literature support.
